# Exploring the social accountability challenges of nursing education system in Iran

**DOI:** 10.1186/s12912-022-01157-w

**Published:** 2023-01-06

**Authors:** Ebrahim Ezzati, Shahram Molavynejad, Amir Jalali, Mohammad-Ali Cheraghi, Simin Jahani, Dariush Rokhafroz

**Affiliations:** 1 Student Research Committee, School of Nursing and Midwifery, Ahvaz Jundishapur University of Medical Sciences, Ahvaz, Iran; 2 Nursing Care Research Center in Chronic Diseases, School of Nursing and Midwifery, Ahvaz Jundishapur University of Medical Sciences, Ahvaz, Iran; 3 Department of Psychiatric Nursing, School of Nursing and Midwifery, Kermanshah University of Medical Sciences, Kermanshah, Iran; 4 Department of Nursing Management, School of Nursing and Midwifery, Tehran University of Medical Sciences, Health Sciences Phenomenology Association, Ministry of Health and Medical Education, Tehran, Iran

**Keywords:** Nursing education, Social accountability, Challenges, Community needs, Nursing Education System

## Abstract

**Background:**

Nursing education in Iran is one of the disciplines of medical sciences and it needs a design tailored to the community needs in terms of theoretical and clinical approaches. This system is currently faced with various challenges. This study aims to explore the challenges of Iranian nursing education system to address community needs.

**Methods:**

A study was carried out through an exploratory descriptive qualitative design with content analysis method. In-depth semi-structured interviews were conducted with 21 participants from the nursing society, selected through purposive sampling. The interviews were continued until data saturation. Data analysis was performed simultaneous with data collection by using Graneheim & Lundman approach.

**Results:**

Based on the interviews and simultaneous analyses, a total of 471 codes, 14 subcategories, six main categories, and two themes were extracted. The first theme, “system structure," consisted of three categories: "the need for ongoing revision of curriculum," "the need to recruit qualified students," and "the need for a proportionate educational environment." The second theme was “the education process” with three categories "the need for purposive educational design," "the need for purposive monitoring and feedback," and "the need for appropriate and early interaction with the community." The participants emphasized the continuous revision of the educational curriculum based on the current needs of the community and community-based nursing education.

**Conclusions:**

In general, the results showed that Iranian nursing education system is faced with many challenges in the educational system structure and processes. It is necessary to make appropriate plans to enhance the status of the educational system structure and develop educational designs to address community needs using a hospital/community-based approach.

## Introduction

As a science that focuses on human healthcare, nursing is committed to promoting health standards and making efforts to address the care needs of patients and the community [1]. The nursing system in every country is the frontline of providing healthcare services [2] and the commitment to the health of people and the community is one of its main duties [3]. Educational systems are complex and consist of individuals, facilities, educational cultures, networks, institutions, identities, and relationships [4]. Educational systems, nowadays, must be accountable to the needs of society [5].

Social accountability is defined as the accountability of individuals, groups, or institutions to the members of a society or an organization by accepting the consequences and results of their responsibilities [6]. Social accountability in the health system and the related disciplines, including nursing, is formed by rational policy-making and effective interventions in the fields of education, care, and research and continuous evaluation and promotion of community health [7]. The system prepares graduates who want and are able to serve their community [8].

According to the social accountability approach, the nursing discipline should be responsible for the quality of care it provides to the community and be able to address people’s care needs by adopting proper policies and strategies [9]. In contrast, poor social accountability can reduce nurses’ scientific and practical ability with regard to the community’s care needs and negatively impact the quality of nursing services, the community’s attitude towards the nursing profession, and public satisfaction with its services [7, 9]. This makes the health care system inaccessible for a large group of people [10].

However, the Iranian nursing system is faced with challenges in terms of social accountability and accountable nursing such as the nursing shortage [11], the gap between theory and practice, limited political influence of nurses, poor engagement in important organizational and national health policies, ignoring nurses’ capabilities in maintaining community health, and nurses limited presence in the community [11–13]. Launching specialty disciplines in nursing master's programs, paying more attention to clinical services, and community-based nursing are among the important measures in the field of social accountability in the nursing profession [13].

Social accountability, in the Iranian nursing system, is considered as a new paradigm in health and cultural change and it is necessary to be studied and understood further in various disciplines including nursing [14]. However, despite the importance of the issue, social accountability in the field of nursing is ignored and there are ambiguities in this regard [15]. The approaches to the nursing system’s social accountability and its different dimensions should be further studied by conducting more quantitative or qualitative research [16, 17].

Among various research approaches, qualitative research can play an effective role in explaining complicated, ambiguous, and unknown areas of phenomena, and lead to the clarification of the why and how of the phenomena of which we have little knowledge [18]. Therefore, this qualitative study was conducted using on an exploratory descriptive qualitative design using content analysis method in order to explore the challenges of the Iranian nursing education system to address community needs.

## Method

### Design

The study was based on an exploratory descriptive qualitative design using content analysis method [19] between May 2020 and December 2021. Qualitative approaches have an explorative nature and enable researchers to explore the complexity of phenomena happening to the healthcare providers, policy-makers, and clients [20].

### Setting

The research environment included hospitals, nursing schools, centers, universities and faculties. Nursing programs in Iran include BSc, MSc, and PhD programs. In the master’s programs, students receive training on various disciplines including pediatrics, internal medicine and surgery, psychiatric nursing, community health, nursing management, rehabilitation nursing, military nursing, and ICU and NICU nursing. In the PhD program, the specialty is determined based on the dissertation topic. Currently, there are 80 state-run nursing schools and 84 nursing schools run by the Islamic Azad University (a Non-governmental university) nationwide training students in nursing bachelor's degree program. Among these schools, 45 (38 governmental and seven Azad University schools) offer Master's programs in addition to Bachelor's degree programs, and 21 (20 governmental and 1 Azad University schools) offer Bachelor's degree, Master's degree, and PhD degree programs. This study was conducted in 12 government colleges and institutions and one non-government institution in the north, center, west, north, southwest and south-central regions of Iran. The inclusion criteria were the age of nursing schools and presence of outstanding nursing professors. The selection was based on a snowball method.

### Participants

The research population consisted of nurses, policymakers, board certified nurses, nursing education and clinical managers, and the faculty members of nursing schools (All participants held at least a Bachelor's degree in nursing). The final research participants consisted of 21 individuals, 11 of them were male and 10 were female (Table 1). All interviewees were members of the nursing community and had a nursing-related education, including 10 nursing professors, four senior and middle nursing managers of hospitals, and four graduates of different programs with clinical jobs. All of participants selected through purposive sampling. Moreover, three more participants were selected through snowball sampling, consisting of a nurse working in community-based centers, a nursing professor, and a nursing PhD student with experience in clinical practice. Finally, data saturation was achieved after the 21^th^ interview. At the end of each interview, the participants were asked to introduce other potential participants for further interviews. Maximum variation in the participants’ selection was taken into account so that the participants were selected from different institutions with diverse work history, experiences, and gender.

**Table 1:** Characteristics of participants in the research

Participants	Job position	Education level	Gender	Job history (year)
P1	Clinical Nurse	BSc in nursing	Female	24
P2	Clinical Nurse	BSc in nursing	Female	17
P3	Home care nurse	MSc in Medical surgical nursing	Male	4
P4	Nursing faculty member	MSc in community health nursing	Female	11
P5	Nursing faculty member	PhD in Nursing	Male	23
P6	Quality improvement office expert	MSc in Medical surgical nursing	Female	19
P7	Community oriented center	MSc in Medical surgical nursing	Female	17
P8	Nursing Manager	MSc in critical care nursing	Male	16
P9	Nursing Manager	MSc in Medical surgical nursing	Female	24
P10	Nursing Manager	MSc in Medical surgical nursing	Male	20
P11	Nursing faculty member	PhD in Nursing	Male	28
P12	Nursing faculty member	MSc in psychiatric Nursing	Male	22
P13	Nursing faculty member	MSc in nursing management	Male	11
P14	Nursing faculty member	PhD in Nursing	Male	27
P15	Nursing faculty member	PhD in Nursing	Female	25
P16	Nursing faculty member	PhD in Nursing	Female	26
p17	Nursing faculty member	MSc in community health nursing	Male	18
P18	Nursing faculty member	PhD in Nursing	Female	19
P19	Clinical Nurse	MSc in Medical surgical nursing	Male	18
P20	Nursing faculty member	PhD in Nursing	Male	21
P21	Nursing students ( PhD candidate)	MSc in Nursing	Female	1.5

#### Inclusion and exclusion criteria

The inclusion criteria consisted of willingness to participate in the study, having knowledge of and experience in the topic under study, the ability to speak, and consent to participate in the interviews. In addition, the exclusion criteria consisted of withdrawal from the study or not allowing the researchers to record the conversations. neither of which happened during the case study.

### Data collection

The data was mainly collected through in-depth semi-structured interviews using open-ended questions. At first, two sample interviews were conducted by the interviewer and reviewed by the research team who had sufficient skills in conducting qualitative research. The guide questions were modified based on the findings. These two interviews were not used in data analysis. The main interviews with the participants were conducted face to face to the convenience of the participants at their workplace. The interviews were conducted by a researcher (the first author) who had completed a full course of qualitative research and voice recorded with the consent of the participants. In addition, memoirs were written and notes were taken to record body language, including the tone of voice, pronunciation of words, laughter, and pauses of the participants. The mean duration of the interviews in each session was 50 to 70 min depending on the subjects’ tolerance. Two of the participants, one nursing professor and one senior nursing manager, participated in two interviews and other participants took part in only one interview. During the interview, guide questions were also used to facilitate data collection. At first, the interviewer would ask general questions and greet the interviewees as a warm-up, then start the interview with the main question “What is your opinion regarding social accountability and nursing education?” Then the interviews would be conducted based on the guide questions.

The guide questions:

- 1. How do you see the current nursing education system in Iranian universities? Can you share your experience?
- 2. How are community needs addressed in the nursing education system structure?
- 3. What are the problems of the current nursing education system to address community needs?
- 4. What is your opinion about the needs or the deficiencies of the nursing education system in addressing community needs?

During the interview, based on the participants’ answers, more open-ended questions were asked to clarify the details of their answers. If the participants started to speak freely, the researcher would direct the interview to further clarify the phenomenon under study by asking the probing question at the appropriate time. Furthermore, the guide questions were reviewed and approved by six researchers, four with PhDs in Nursing and two with PhDs in Medical Education.

### Data analysis

In order to analyze the data, a qualitative approach was used based on Graneheim & Lundman qualitative content analysis method [20]. The qualitative analysis was done with completion of each interview, i.e. after each interview, the collected data would be analyzed and then the second interview would be conducted. To analyze the interviews, the first author did the initial coding of the interview, then a three-member team, consisting of the first, second, and third authors summarized the analysis of each interview, and pointed out the shortcomings of the interview to the first author (the interviewer). All the interviews were analyzed in this way, and after the completion of the interviews, the preliminary classes and sub-classes were formed from the second interview onwards.

To analyze each interview, after jotting down the interviews, the texts were reviewed several times (immersion in data) to achieve a general perception of the different aspects of the experiences and the perspectives. The sentences were then converted into units of meaning as parts of sentences or paragraphs, and the initial codes were obtained using the words close to the participants’ statements. Finally, the codes were categorized based on the differences and the similarities. Then they were classified based on similar meanings and the connections and the consistency among them. Thus, the categories were placed together based on meaningful conceptual patterns. By realizing the connections in the data, the themes emerged. At this stage, MAXQDA software version 18 was used for data management.

### Rigor

Credibility, dependability, confirmability, and transformability were assessed to approve the trustworthiness of the findings [21].

To confirm the acceptability of the data, methods such as long-term involvement with the study and data and integration in the study such as maximum variation in the participant’s selection in terms of workplace, work experience, and gender were used. In addition, the research colleagues (peer check) and study participants (member check) checked the codes and classes.

To determine the reliability of the data, auditing method was used in this study. So that all research steps and details of the process, concepts, and results were clearly written. In addition, MAXQDA software V.18 was used to make sure that other researchers could follow and audit the data.

To determine the verifiability, the researcher tried to abandon her presuppositions about the desired phenomenon and the results (bracketing). In addition, the researcher tried to explain the different stages of the research and all the activities in detail so that other people can do an external audit of the subject by reading these writings.

To confirm the transferability in this study, the desired phenomenon was briefly examined and all the characteristics related to the cultural and contextual situations of the participants in the study were explained.

## Results

A total of 21 interviews were conducted with 21 subjects. Based on the interviews and the simultaneous analyses, two themes, six main categories, 14 subcategories, and 471 codes were extracted. Table 2 displays the categories and subcategories obtained through the interviews.

**Table 2:** Categories and subcategories

Subcategories	Categories	Theme
Ignoring the Current Needs of the Community	The Need for Ongoing Revision of Curriculum	System structure
The Need for a Community-Based Curriculum		
Focusing on Appropriate Community and Hospital-Based Practice		
Having Interest in the Career	The Need to Recruit Qualified Students	
Potential Talents and Abilities		
Capable and Committed Human Resources	The Need for a Proportionate Educational Environment	
Clinical Ward Space Proportionate to the Number of Students		
Attending Community-Based Care Centers		
Making Theoretical Education Target-Oriented	The Need for Purposive Educational Design	The Educational Process
Client-Centered Clinical Education		
The Standardization of Theoretical and Clinical Evaluation	The Need for Purposive Monitoring and Feedback	
Proportionate Monitoring and Feedback		
Focusing on Necessary Clinical services	The Need for Appropriate and Early Interaction with the Community	
Early Interaction with the Community		

### Describing categories and subcategories

#### System structures

It was one of the main themes of the study, consisting of three categories and eight subcategories as follows:

##### The need for ongoing revision of curriculum

This category contains three subcategories and it is one of the most important concepts extracted, which can be the system structures for the education of nursing students tailored to the current community needs.

##### Ignoring the current needs of the community

This subcategory was formed based on the data derived from the participants’ statements, emphasizing the need to focus on the current needs of the community, emerging diseases, and hospital- and-community-based services. The following are the participants’ quotes on the matter:

“Today, people need help for chronic and emerging diseases. However, due to COVID-19 pandemic, chronic diseases have been ignored to a great extent, and the topics about the levels of prevention have been disregarded” (P12).

“*We studied lots of stuff in the program, but unfortunately the fact is that most of the materials cannot help us much in practice” (P1).*

##### The need for a community-based curriculum

By analyzing the participants’ statements, it can be inferred that it is necessary to arrange the nursing education curriculum at different degrees, especially at undergraduate programs, using hospital/community-based perspectives, along with ongoing revisions. The following are some examples in this regard: “*The nursing education curriculum is well-formulated. However, for developing practical courses in different settings, in addition to training and apprenticeship programs at hospitals, different areas of community-based services should also be considered*” *(P14).*

“A developed curriculum should target issues based on the current needs of the community. Hospital, community based, and practical training programs must be offered in the revisions and community health courses should be presented in a more practical manner” (P15).

##### Focusing on appropriate community and hospital-based practice

The majority of the participants believed that the nursing education curriculum at different levels, especially at the nursing undergraduate program, was formulated based on the hospital-based nursing services. The community-based framework only exists in community health courses, and in community health and geriatric nursing disciplines, at the master’s level. However, due to the country’s need for nursing experts’ working at hospitals, and even community nursing centers, it is necessary to include hospital-and-community-based materials proportionally while designing the curriculum. Participants said in this regard:

“The nursing program, courses, and even training and apprenticeship programs are focused on the nurse's hospital activities. It was only during the community health nursing course that we received little training on the level one of prevention services,” (P2).

“*Graduated nurses who enter hospitals all have a hospital-based perspective and hospital services are the centerpiece of all their activities and even their paradigm, with practically no tendency to provide community-based services” (P24).*

##### The need to recruit qualified students

The data obtained from the interviews showed that the number of qualified students and learners was one of the effective concepts in explaining the challenges of Iranian nursing education system. This category consisted of the following two subcategories:

##### Having interest in the career

Considering the occupational opportunities for other disciplines and the large demand for nursing in the job market, most students in this discipline are not really interested in it and their only motive is the good job opportunity. The following are some examples in this regard:

“Currently, the only criteria for admitting students in the program is the admission exam, and considering the good job opportunities for nurses, many students who choose this discipline only do this for the good job opportunity” (P16).

“*Students are often not interested in nursing and do not have the motive to study or even learn nursing health care. This lack of interest is more common in male students compared to the female ones*" *(P18).*

##### Potential talents and abilities

The participants emphasized that the students’ potential talents and abilities was an important factor in training nurses.

“*Many freshman students are very intelligent and talented. But unfortunately, most of them focus their efforts on studying the materials needed to apply for non-nursing disciplines at higher levels, instead of focusing on learning and empowering themselves for clinical practice, and practically strive for higher degrees” (P11).*

##### The need for a proportionate educational environment

Another category extracted in this study was *appropriate educational environment*, consisting of the following three subcategories:

##### Capable and committed humanresources

In light of considering the significance of clinical education in the field of nursing, the participants believed that the presence of skilled professors and nurses was of great importance.

“*There are a few good professors in the faculty and the rest are the graduated students with no clinical experience. They have not even worked one on-call night shif*t” *(P21).*

“*Many novice professors lack the skill and even the interest in teaching the students*” *(P3).*

##### Clinical ward space proportionate to the number of students

The participants believed that due to the large number of students in clinical wards from different disciplines, clinical education space in inpatient and outpatient wards is not proportionate to the number of students. The following are some statements in this regard:

“*There were too many medical and nursing students from different faculties. Sometimes, during the training courses, there were even fewer patients than the students*” *(P16).*

“*There are too many students in some clinical courses and we cannot teach the skills they need to all of them” (P13).*

“*Unfortunately, our hospital wards are not educational. The nurses do not acknowledge students at all. In many wards, head nurses are not willing to see students in wards, and keep saying that, students only increase the workload and they have to monitor the students*.*” (P5).*

##### Attending community-basedcare centers

It was found that attendance at community-based care centers was too low. Further planning and coordination is required to offer training and apprenticeship programs at community-based care centers. The following are some statements in this regard: 

“*Educational content in the field of community-based care is theoretical, and the students do not practically get acquainted with the concept of community-based care in a clinical manner during the program” (P7).*

“*There is no proper planning within our treatment and educational system for the increased presence of nurses in the community. Therefore, in practice, in the educational programs for students, attending community-based care centers is limited to public clinics” (P12).*

#### The educational process

One of the important themes was nursing education challenges consisted of three categories and six subcategories:

##### The need for purposive educational design

This category was also one of the important concepts in the study, which was mentioned both directly and indirectly by the participants and consisted of the following two subcategories:

##### Making theoretical education target-oriented

The participants believed that in the educational design of nursing education courses at different programs, theoretical education should be purposive and in line with community-and-hospital-based clinical education. The following are some examples in this regard:

“Due to the large number of students in the classroom, in most cases, theoretical courses are limited to lectures” (P11).

“*Theoretical lessons should be presented using real and objective cases in an understandable manner for students” (P20).*

##### Client-centered clinical education

One of the important topics in educating nursing students is to offer clinical courses as apprenticeship and training programs in a practical setting. All the participants stated that the clients and their needs should be considered in clinical training, and the student should realize that the most important issue in clinical practice is the clients’ needs and their preferences.

“It is really important in clinical education to properly interact with patients, pay attention to them and accept them and focus on their problems and culture” (P15).

##### The need for purposive monitoring and feedback

This concept was one of the main categories consisting of the following two subcategories that can be effective in improving students’ clinical practice:

##### The standardization of theoretical and clinical evaluation

According to the participants, it was revealed that theoretical and clinical evaluation approaches need to be redesigned and standardized based on modern evaluation approaches. In this regard, adopting modern methods of clinical evaluation is of great importance. The following are some examples in this regard:

“There is no proper and purposive clinical evaluation in many apprenticeship and training programs. Sometimes, the evaluation is done through a checklists or asking a few questions,” (P14).

“*There was no certain plan in apprenticeship and training programs. The instructor only checked to see if we read patients’ medical records, administered their drugs, and checked the vital signs. There was no evaluation. The questions that were asked during the course were considered as evaluation and all the students received similar marks with slight differences*” *(P21).*

##### Proportionate monitoring and feedback

Another important subcategory was proportionate monitoring and feedback. The participants mentioned the need for the planning and implementation of standard and appropriate monitoring and evaluation through providing early feedback. They considered this topic as one of the necessities of the current nursing education program.

“Unfortunately, no regular and planned evaluation is done in apprenticeship and training programs and sometimes, evaluations are left incomplete. In most cases, no proper feedback is provided to the students” (P20).

“*In the nursing education curriculum, appropriate logbooks and recommendations for evaluation are presented but, in most cases, the items listed in the logbook are not fully and seriously implemented, and there is no feedback, either*,” *(P13).*

##### The need for an appropriate and early interaction with the community

This category was derived from the concept of focusing on the necessary clinical services and the early interaction with the community.

##### Focusing on necessary clinical services

The participants stated that the current hospital-and-community-based nursing care required by the community must be included in the theoretical and clinical education of students:

“There is not enough time to deal with all the required cases during apprenticeship, and we have to cover most of the cases through videos and homework” (P17).

*“In general, the professors only mentioned the cases and the problems related to hospital wards. We did not see many of the cases, and community health nursing training in clinics was limited to vaccinations and injections, and visiting a few factories. We did not experience community-based care” (P18*).

##### Early interaction with the community

According to the participants, the students should enter clinical fields at hospital and in the community as soon as possible, right after entering the nursing program. This helps them to better understand the community needs for nursing services and properly interact with the community. The following is an example in this regard:

“*If students enter hospitals and home care centers at the beginning of their education, they will spend more time doing clinical practice and interacting with people. In this way they can better understand people’s needs and prepare themselves to address these needs*,” *(P4).*

## Discussion

An exploratory descriptive qualitative design using content analysis method was carried out to explore the challenges of the Iranian nursing education system in order to address the community needs.

In order to respond to the needs of the society, the Iranian nursing education system deals with two themes including system structure and educational process. In a similar study, an appropriate educational system structure including competent instructors and proper educational processes and design was considered as the foundation and the necessity of nursing education [22]. Another study also referred to developing system structure and making changes tailored to community needs in educational processes as an important factor to reform nursing education in the future [23]. Therefore, it can be said that educational system structure and processes are the main components of educational systems.

The results showed that the nursing curriculum should be regularly revised based on the community needs. One of the important categories was ignoring community needs in the nursing curriculum. The main requirements and needs in the nursing discipline are paying attention to community needs and providing services accordingly. Addressing community needs is one of the main priorities of nursing systems [24, 25]. There is a need for a very systematic and continuous revision of the curriculum based on the present educational needs of the community [26]. It can be inferred that as a result of the rapid growth of publishing papers in the field of nursing and medical sciences and the expansion of knowledge in this area [8, 27] as well as the changing needs in community and expectations of nursing and medical services [28, 29], it is necessary for the nursing discipline to continuously update the curriculum to keep it purposive.

Another important finding was the topic of qualified students. Being interested in nursing and having the potential abilities and talent is essential to enter this discipline. Some studies have stated that in order to succeed in this discipline, in addition to being interested and having potential abilities and talent, nurses should try to introduce their profession and its position to the public through enhancing their attitudes [27, 30, 31]. In one of these studies, the researchers discovered that it was necessary to improve both students and nurses’ attitudes toward the values ​​of nursing discipline [31]. One of the important measures is to select interested and talented students with the potential ability to enter this field, and to make plans and take measures to improve their professional values and attitudes through in-service training, during the program and service.

A proportionate educational environment was another finding. The educational environment is one of the major requirements of the educational system, in terms of the presence of competent and committed faculty members, educational clinical wards proportionate to the number of the students, as well as community care centers. Moreover, it can be effective in making the education process accountable to the needs of the society. Universities should make plans to optimize the educational environment. Many studies have emphasized the importance of the educational environment, the evaluation of the situation, and the optimization of the learning environment in nursing education [32–34]. The accreditation of educational environments and especially the qualitative and quantitative status of professors, the clinical and theoretical education environment and equipment, and educational processes is one of the appropriate ways to empower disciplines to address the community needs [35]. It can be concluded that the educational environment consisting of human resources, clinical learning environments, and community-based care clinics is one of the requirements of education in the nursing system. The optimization of which is one of the most efficient measures to improve the Iranian nursing education system.

Given the continuous changes in the society’s needs in the health sector the Iranian nursing education system needs to be revised or even educationally redesigned to highlight the provision of theoretical and clinical education with an emphasis on the community needs and community-based care programs. Studies have shown that a community-based curriculum improves professional skills, communication skills, self-confidence, knowledge and awareness, critical thinking skills, and teamwork skills among nursing graduates [25]. In another study, the authors showed that the community-based participatory educational programs had impacts on addressing the communication and participatory issues of nursing and midwifery students, and significantly improved their self-confidence and promoted their learning [36]. To explain the results, it can be concluded that hospital and community-based clinical care centers play an important role in the education of nursing students. Therefore, it is necessary to include students’ presence in hospital and community clinical environments in the curriculum.

The results of the study also showed that monitoring and providing feedback in educational activities and the use of appropriate and standard educational evaluation methods in theoretical and clinical education were essential for nursing education and needed further improvement. Providing appropriate feedback to and purposive monitoring of students based on community-based curriculum can empower the students and make them feel responsible for the needs of the community [37]. Currently, monitoring and evaluation in Iranian nursing schools is based on teacher-made exams and it is necessary to enhance evaluation in schools based on standard and advanced evaluation approaches [38]. Thus, the use of mixed-method clinical-based evaluation approaches could be really useful in evaluating the clinical competence of nursing students [39]. In explaining the results, it can be said that the current nursing education system needs to promote and improve evaluation approaches, especially in clinical practice. To this end, feedback should be provided in clinical and theoretical evaluations along with using novel and efficient evaluation approaches.

The nursing education system needs to improve the interaction with the community. More focus is needed on clinical services and community-based care, and early interaction with the community should be a priority in the education system. Various studies have also emphasized community-based clinical education [8, 36] and having appropriate community interaction [25]. It can be concluded that in order to address the community needs, it is necessary to make changes in the nursing curriculum in order to enhance students’ interaction with the community, especially in community-based care centers. In addition to hospital-based clinical training sessions, community-based health care nursing training are essential.

### Strengths and limitations

One of the advantages of this study is adopting a qualitative approach in a real environment that provides a true representation of the process under study. Furthermore, conducting interviews with a wide variety of nursing professions, nursing professors, and students is noteworthy. Unfortunately, due to the COVID-19 pandemic, the participants were not willing to be interviewed for nearly a year. Therefore, the interviews were conducted after a decline in coronavirus cases and, at times, when the city of Kermanshah was in better conditions. Sampling and conducting the interviews took more than one year due to the community conditions. The study was prolonged inevitably, due to the closure of facilities and universities, as well as the participants’ reluctance to conduct interviews. In addition, considering the limitations of quantitative studies and the need to describe the challenges in the Iranian nursing education system in a real environment, a qualitative method was used in this study. Therefore, given the ongoing changes in the educational system and the needs of the health sector in the society, similar studies are needed in the future.

## Conclusion

The Iranian nursing education system is faced with many challenges in the field of educational system structure and educational process. In this regard, the need for revising the curriculum, more attention to the ever-changing community needs, and adopting standard and target-oriented educational approaches in the clinical and theoretical areas are all considered as the requirements of the Iranian nursing education system to cover the needs of society.

## Data Availability

The data that support the findings of this study are available from Ebrahim Ezzati but restrictions apply to the availability of these data, which were used under license for the current study, and so are not publicly available. Data are however available from the authors upon reasonable request and with permission of Ebrahim Ezzati.
